# Genome Sequence of Azospirillum brasilense REC3, Isolated from Strawberry Plants

**DOI:** 10.1128/genomeA.00089-18

**Published:** 2018-02-22

**Authors:** Cecilia Alejandra Fontana, Sergio Miguel Salazar, Daniela Bassi, Edoardo Puglisi, Nadia Lovaisa, Lucía Mercedes Toffoli, Raúl Pedraza, Pier Sandro Cocconcelli

**Affiliations:** a INTA EEA Famaillá, Tucumán, Argentina; b Facultad de Agronomía y Zootecnia, Universidad Nacional de Tucumán, Tucumán, Argentina; c Istituto di Microbiologia, Università Cattolica del Saco Cuore, Cremona-Picenza, Italy

## Abstract

The genome sequence of a plant growth-promoting bacterium and biocontrol agent, Azospirillum brasilense REC3, isolated from strawberry roots, is reported here. The A. brasilense REC3 total genome contains 7,229,924 bp and has a G+C content of 68.7 mol%.

## GENOME ANNOUNCEMENT

Azospirillum brasilense REC3 was isolated from inner-root tissue samples of strawberry plants cultivated in Tucumán, Argentina (1). This strain exhibits the main characteristics that define plant growth-promoting bacteria, namely, nitrogen fixation and the production of siderophores and indoles, and contributes to the mineral nutrition of strawberry plants, reinforcing their physiological and cellular characteristics. Previous studies reported that, among other characteristics, A. brasilense REC3 enhanced levels of soluble phenolic compounds, reduced lipid peroxidation, and promoted the upregulation of strawberry genes involved in bacterial recognition and defense and H_2_O_2_ depuration (2–4). By using a green fluorescent protein-tagged gene, it was demonstrated that REC3 is capable of colonizing strawberry roots, promoting their growth under controlled environmental and field conditions, and colonizing newborn plants via stolons (5–7). A. brasilense REC3 is also an active participant in the systemic protection of strawberry plants against anthracnose disease caused by Colletotrichum acutatum (8). Moreover, besides its association with strawberry plants, A. brasilense REC3 has been shown to improve grain yields of rain-fed rice crops (9).

In this study, the whole genome of A. brasilense REC3 was sequenced using the Illumina HiSeq platform. The total size was 7,229,924 bp, representing a genome coverage of approximately 99.9×. Quality-filtered reads were assembled using Velvet version 1.1.04 (10), which generated 137 contigs. The contigs were ordered against A. brasilense Az39 (11) using progressive MAUVE multiple-genome alignment software (12), which identified 103 contigs coming from the plasmid DNA. The maximum contig size for the plasmid DNA was 333,173 bp, confirming the presence of large plasmids in A. brasilense REC3. Annotation with the Rapid Annotations using Subsystems Technology (RAST) server (13) revealed that the total genome contains approximately 6,526 coding genes, 84 tRNAs, and 513 subsystems. The genome of this strain has a high G+C content (68.7%). Putative functions of the coding genes related to plant growth promotion, including nitrogen fixation, phytohormone and plant growth-regulator biosynthesis, biofilm formation, and type I, II, and VI secretion systems, were automatically identified using the RAST server.

Further comparative genome analyses with other strains from different sources are in progress, with the goal of investigating the specific mechanisms of Azospirillum plant-pathogen interactions to elucidate the potential of this microorganism to be used as a biocontrol agent.

### Accession number(s).

This whole-genome shotgun project has been deposited at DDBJ/EMBL/GenBank under accession number POWG00000000. The version described in this paper is the first version, POWG01000000.
